# Clinical efficacy of transverse preputial island flap urethroplasty for single-stage correction of proximal hypospadias: a single-centre experience in Chinese patients

**DOI:** 10.1186/s12894-020-00686-3

**Published:** 2020-08-05

**Authors:** Xu Cui, Yuanbin He, Wenhua Huang, Liu Chen, Yunjin Wang, Chaoming Zhou

**Affiliations:** Department of Pediatric Surgery, Fujian Maternity and Child Health Hospital, Affiliated Hospital of Fujian Medical University, Fuzhou, 350001 People’s Republic of China

**Keywords:** Proximal hypospadias, Transverse preputial island flap urethroplasty, Postoperative complications, Satisfaction with penis appearance

## Abstract

**Background:**

This study was designed to summarize the clinical outcomes of transverse preputial island flap urethroplasty for single-stage correction of proximal hypospadias in our hospital.

**Method:**

This study retrospectively analysed the clinical data, including the preoperative general information, intraoperative and postoperative data, and follow-up data, of 155 children with proximal hypospadias who were admitted to our hospital from January 2009 to January 2019.

**Results:**

During follow-up, a total of 92 postoperative complications occurred, and 41 patients underwent reoperation. There were 49 patients with urinary fistula, 26 patients with urethral stricture, 9 patients with urethral diverticulum and 8 patients with urinary tract infection. Regarding the family members’ satisfaction with the cosmetic appearance of the penis, the satisfaction rate with the urinary meatus was 85.2%, the satisfaction rate with the glans appearance was 87.7%, the satisfaction rate with the the appearance of the foreskin of the penis was 92.3%, and the satisfaction rate with the overall penis shape was 89.0%.

**Conclusion:**

Proximal hypospadias is a serious condition that is often combined with severe chordee, and transverse preputial island flap urethroplasty for single-stage correction is an effective surgical procedure for treating this condition.

## Background

Hypospadias is one of the most common congenital urinary malformations in children [1]. Proximal hypospadias refers to a urinary meatus that is located at the shaft of the penis near the scrotum, the junction of the penis and scrotum, or the perineal area [2]. Hypospadias is a serious condition, and surgery is the only treatment for proximal hypospadias, although it is associated with a difficult technique and high postoperative complication rate. Despite decades of surgical improvement, there is currently no single procedure that can perfectly solve all of the problems associated with the proximal type of hypospadias [3]. A high incidence of postoperative complications is still a major challenge for paediatric urologists.

At present, there are two types of operations for proximal hypospadias, single or two-stage surgery, and the choice of operation is still controversial. Long CJ et al. found that single-stage surgery led to a higher incidence of postoperative complications than two-stage surgery [4]. However, recent studies have also shown that the complication rate of two-stage surgery was higher than expected, reaching as high as 30–68%, and with the improvements in surgery technology, single-stage surgery did not lead to an increase in the incidence of postoperative complications and can reduce the number of operations needed [5, 6]. This study retrospectively analysed the clinical data of patients receiving transverse preputial island flap urethroplasty for single-stage correction of proximal hypospadias in our hospital in the past 10 years, and we summarized our clinical experience over these 10 years.

## Methods

This research was approved by the ethics committee of our hospital and strictly abides by the principles of the Helsinki Declaration (the code of ethical approval for scientific research projects, 2019 Ethical Scientific Research Approval No. 2005).

### Patient criteria

**Fig. 1:**
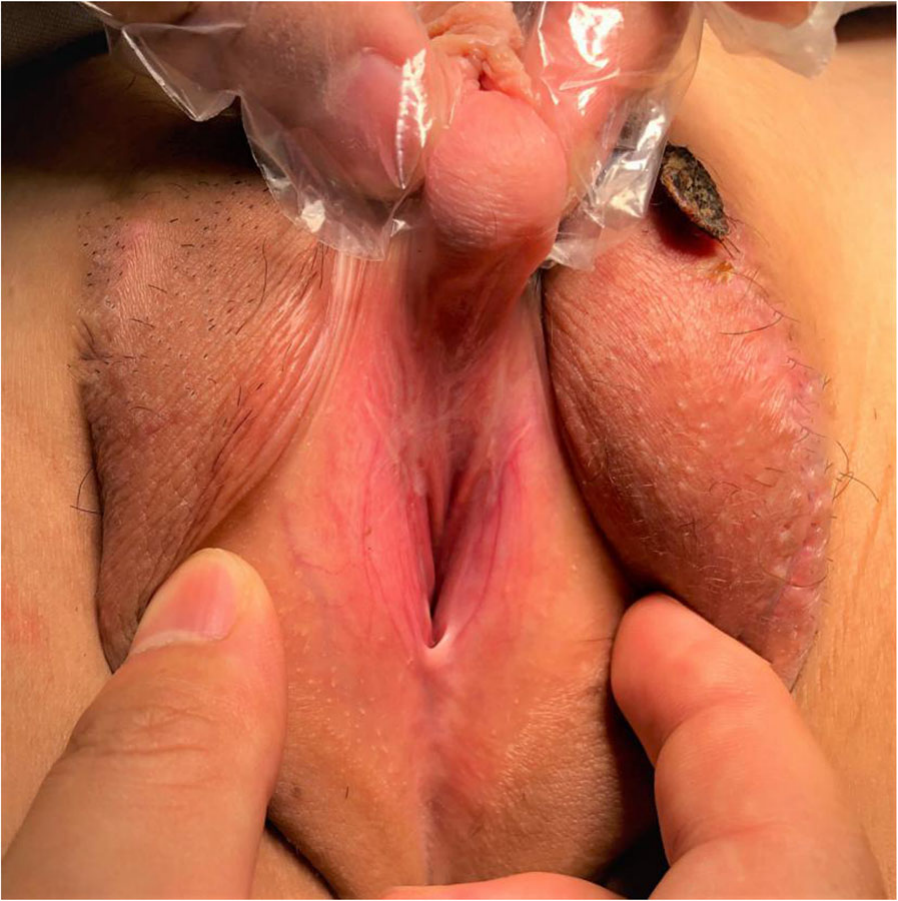
Proximal hypospadias of the perineum

This study retrospectively analysed the clinical data, including the preoperative general information, intraoperative and postoperative data, and follow-up data, of 155 children with proximal hypospadias who were admitted to our hospital from January 2009 to January 2019. According to the patient’s clinical manifestations and physical examination, all patients were diagnosed with proximal hypospadias (Fig. 1), including 50 cases of a urinary meatus in the proximal penile body (32.3%), 84 cases of a urinary meatus in the penile scrotum junction (54.2%), and 21 cases of a urinary meatus in the perineum (13.5%). All patients underwent surgery performed by a paediatric urology chief physician and two attending physicians.

The inclusion criterion was proximal hypospadias, and the exclusion criteria were as follows: 1. other subtypes of hypospadias; 2. complex urogenital malformations and hermaphroditism; 3. need for reoperation; and 4. refusal to undergo the operation or refusal to cooperate during the follow-up period.

### Operative technique

**Fig. 2:**
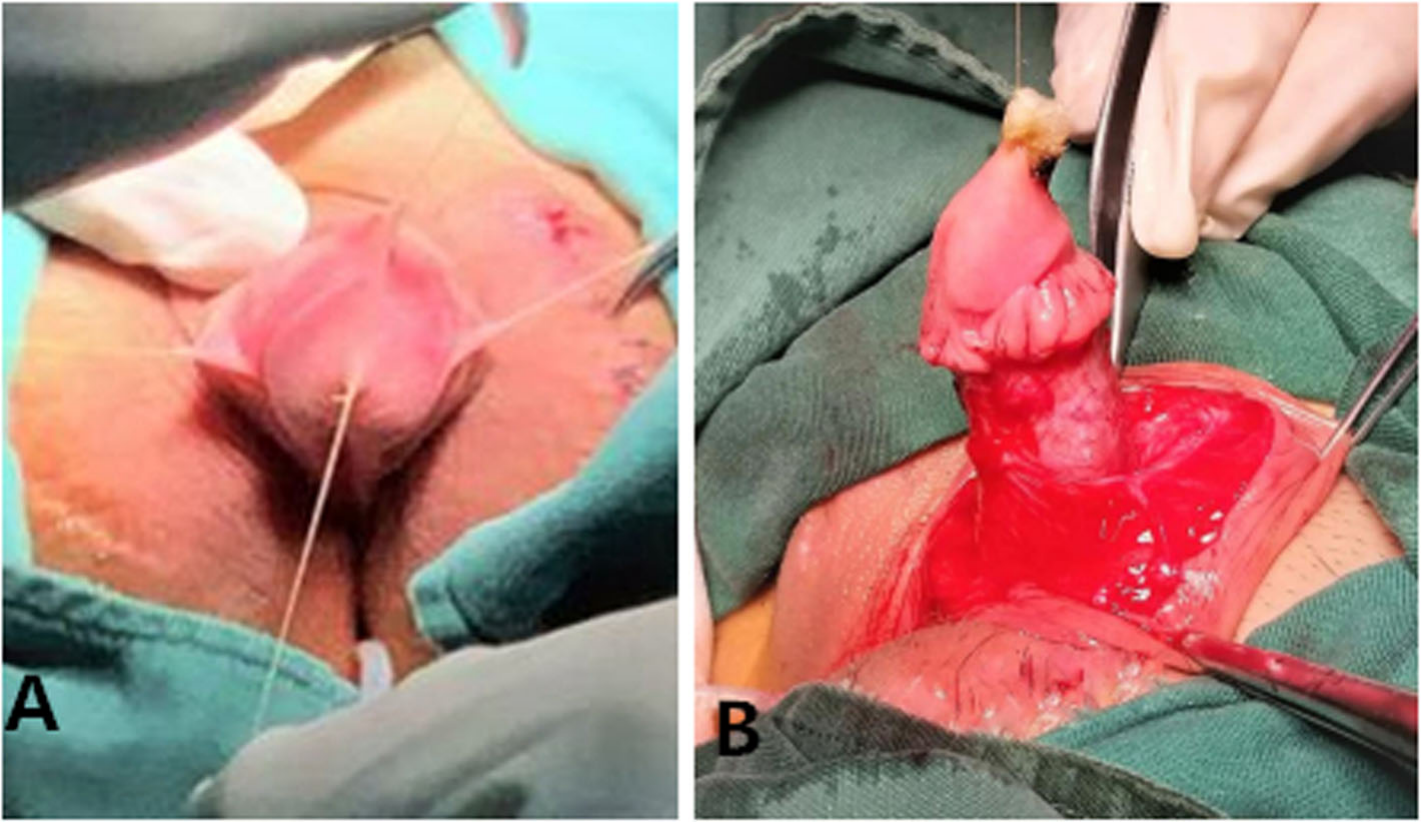
**a** Suspension of the glans and dorsal penis. **b** The fibrous tissue from the ventral side of the corpus cavernosum to the upper edge of the urethral meatus was removed to correct chordee

**Fig. 3:**
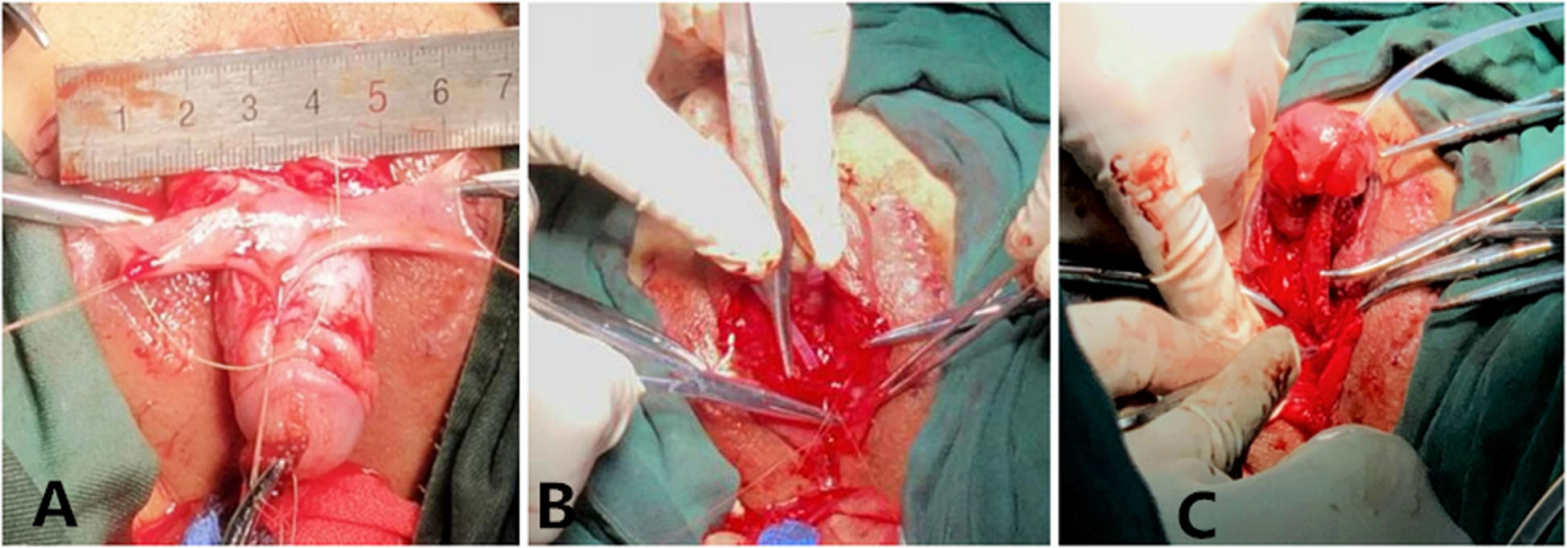
**a** Image of the raised flap. **b** The free vascular pedicle reached to the root of the penis to form a vascularized flap. **c** A new urethral orifice was formed by pulling the newly formed distal urethral tube through penile tissue of a suitable calibre

**Fig. 4:**
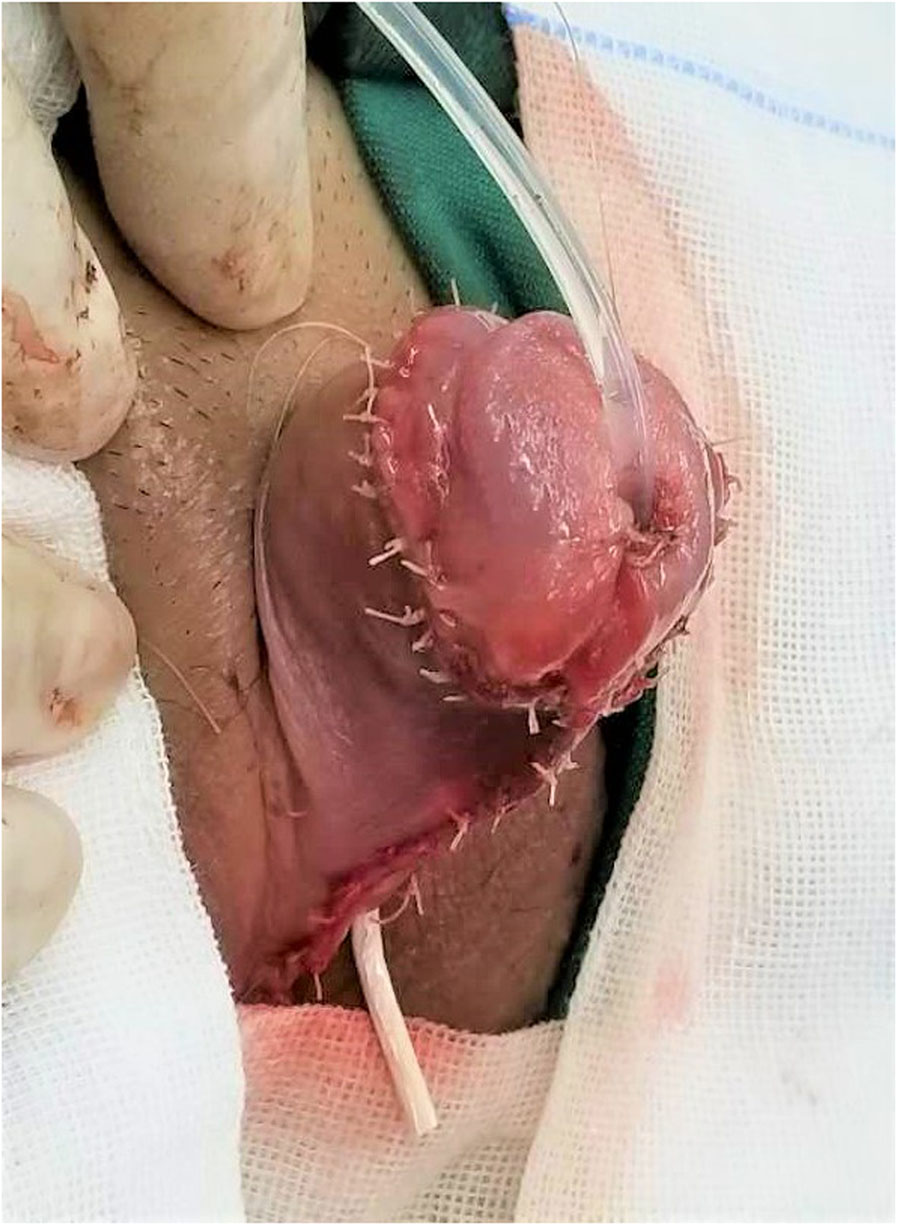
The skin incision was sutured

After anaesthesia, the patients were placed in the supine position with their buttocks slightly raised. First, the glans was suspended with a 5–0 absorbed suture with 1.0 cm between the urethral plate and the back of the glans. Additionally, the dorsal foreskin of the penis was suspended in three points 0.5–0.8 cm from the coronary. Second, the foreskin was injected with saline, and then the penis was degloved. Third, the urethral plate was cut laterally 0.8 cm from the coronary sulcus, and the fibrous tissue of the ventral side of the corpus cavernosum to the upper edge of the urethral meatus was removed to correct chordee (Fig. 2). Fourth, the foreskin was cut at the junction of the inner and outer plates, and the proximal free vascular pedicle was stretched to the root of the penis to form a vascularized flap. Fifth, the vascularized flap was turned to the ventral side parallel to the penis, with a balloon silica catheter serving as the inner stent, and 6–0 absorbable sutures were used to close the vascularized flap. After the urethral orifice was freed and the membranous urethra was excised, the new and old urethras were sutured intermittently with a 6–0 absorbable suture line. Then, the distal end of the urethral tube and the glans were sutured intermittently to form a new urethral orifice by pulling the newly formed distal urethral tube through penile tissue of a suitable calibre (Fig. 3). The edge of the urethral tube was sutured to the ventral cavernous body, and then the subcutaneous tissue and prepuce were sutured. Next, a rubber drainage strip was placed on the same side of the vascular pedicle under the penile skin and exposed via a puncture incision. Then, the skin incision was sutured (Fig. 4). Finally, the penis body was bandaged with Vaseline gauze and an elastic bandage.

### Postoperative course

After the operation, the penis was wrapped with silver ion gauze, and the outer layer was covered with elastic bandage. Pressure was gently applied on the penis dressing to drain fluid from the wound area, and a 0.05% benzalkonium chloride swab was used to disinfect the scrotal incision and the skin around the rubber drainage strip. The external bandage on the penis and the rubber drainage strip were removed 5 days after surgery. The catheter was removed 10–21 d after surgery.

### Follow-up

Follow-up examinations were performed at 2 weeks, 1 month, 3 months, 6 months, and 1 year after discharge, and every other year thereafter. The follow-up visit evaluated the urination ability of the child and the family’s satisfaction with the appearance of the penis. Penile appearance satisfaction after hypospadias repair was measured by the Pediatric Penile Perception Score (PPPS) [7, 8], which includes satisfaction with the urethral meatus, glans appearance, shaft skin, and general appearance. For each aspect, the patients could express satisfaction based on four levels, including very dissatisfied (0 points), dissatisfied (1 point), satisfied (2 points) and very satisfied (3 points), where 2 points and above was considered satisfied. At the 1-year follow-up visit, all parents completed a PPPS questionnaire to assess their satisfaction with the appearance of the penis.

## Results

All 155 patients, aged 6 months to 12 years old, weighing 6.8 kg–55.8 kg, underwent transverse preputial island flap urethroplasty for single-stage correction; there were 50 cases of a urinary meatus in the proximal penile body, 84 cases of a urinary meatus in the penile scrotum junction, and 21 cases of a urinary meatus in the perineum. The median operation time was 2.8 h (2.3 h - 3.9 h), the median length of postoperative hospital stay was 15 d (12 d – 29 d), and the median follow-up time was 5.2 years (3 months − 10 years). A total of 92 (59.3%) postoperative complications occurred, and 41 (26.5%) patients underwent reoperation. There were 49 patients (31.6%) with urinary fistula; among them, 7 healed by themselves, and 42 were cured after reoperation. Urethral stricture occurred in 26 cases (16.8%), of which 22 patients were cured after urethral dilatation, and 4 patients were cured after reoperation. There were 9 patients (5.8%) with urethral diverticulum after operation, all of whom were cured after reoperation. There were 8 patients (5.2%) with urinary tract infection, all of whom were cured by medical treatment. There were no cases of chordee recurrence or infravesical obstruction after surgery (Table 1).

**Table 1:** Demographical and clinical characteristics of the patients in this study

Item	
Number of patients	155
Age (year), median (range)	1.8 years (6 months - 12 years)
Weight (kg), median (range)	11.5 kg (6.8 kg - 55.8 kg)
Location of proximal hypospadias (%)	
Proximal penile body	50 (32.3%)
Penile scrotum junction	80 (54.2%)
Perineum	21 (13.5%)
The operation time (h), median (range)	2.8 h (2.3 h - 3.9 h)
The postoperative hospital stay (d),median (range)	15d (12d - 29d)
The median follow-up tim (year), median (range)	5.2 years (3 months − 10 years)
Postoperative complications (%)	92 (59.3%)
Location of proximal hypospadias Urinary fistula	49 (31.6%)
Urethral stricture	26 (16.8%)
Urethral diverticulum	9 (5.8%)
Urinary tract infection	8 (5.2%)
Infravesical obstruction	0
Recurrence of penile curvature	0
Patients of second operation	41 (26.5%)

The family members’ satisfaction with the cosmetic appearance of the penis were as follows: the satisfaction score for the urinary meatus was 2 (1–3), with a satisfaction rate of 85.2% (132/155); the satisfaction score for the glans appearance was 2 (0–3), with a satisfaction rate of 87.7% (136/155); the satisfaction score for the appearance of the foreskin of the penis was 2 (1–3), with a satisfaction rate of 92.3% (143/155); and the satisfaction score for the overall penis shape was 2 (1–3), with a satisfaction rate of 89.0% (138/155).

## Discussion

Hypospadias is one of the most common congenital structural malformations in the urinary system, with the second highest incidence among all such malformations. According to Springer’s latest global multicentre epidemiological survey, the incidence rate of this condition in Asia has risen to 0.6–110/10,000, and the southeast coast is in a high-incidence area [1]. Proximal hypospadias is a serious type of the condition that is often combined with severe chordee. It is difficult to correct the malformation, and there are still many postoperative complications after surgery. After many years of development, both the operative skills for treating proximal hypospadias and the cure rate have greatly improved [9], but there is no perfect surgical method to avoid postoperative complications [2]. Controversy still exists with regard to the choice of single-stage or two-stage surgery. Many scholars have found that as long as the surgical technique was mature, the postoperative complications of single-stage surgery would not exceed that of two-stage surgery; single-stage surgery can reduce the number of surgeries to one and reduce the burden on the patients and the pain of a second operation. Singal AK et al. reported a summary of 136 patients with proximal hypospadias who underwent transverse preputial onlay island flap urethroplasty for single-stage correction over 7 years, and the results showed that single-stage operations could provide reliable functional results [10]. The study by Emir H et al. showed that the mature technique of single-stage surgery for proximal hypospadias was desirable for achieving good results and recovery satisfaction [11]. Castagnetti M et al. found that single-stage surgery for proximal hypospadias did not increase the overall incidence of postoperative complications compared with two-stage surgery [12]. We used the single-stage transverse preputial island flap urethroplasty method to treat proximal hypospadias. After more than 10 years of development, we have accumulated some experience and have reported this experience as follows.

Urinary fistula is the most common complication after surgery and can occur anywhere in the proximal anastomosis. The cause is related to poor blood supply to the newly formed urethra or poor blood effusion [13–16]. We summarized our recommendations as follows: 1) pay attention to protecting the vascular pedicle of the inner sheath of the foreskin and ensure that the transverse flap is as thick as possible so that the vascular tissue can be preserved. The length of the vascular pedicle is based on the diameter of the penis, and there must be no tension from the root of the vascular pedicle when the flap is transferred. 2) When the newly formed urethra and the original urethra are anastomosed, the marginal skin with poor blood supply at the end of the newly formed urethra must be removed. An oblique anastomosis should be adopted to increase the area of the anastomotic surface and the blood supply of the anastomosis. 3) At the end of surgery, we routinely placed a rubber drainage strip from the outside of the scrotum to the ventral urethral anastomosis of the penis. This tube can fully drain the blood, provide effusion to the penile corpus cavernosum and create good conditions for wound healing.

Urethral stricture is another common complication after surgery for hypospadias [17, 18]. The cause is related to the thickness of the urethral stent tube, local inflammation, scars, etc. [19] Mild urethral stricture can be relieved after multiple urethral dilatations, but a severe urethral stricture requires repeat urethroplasty [20]. Our experience is that the urethra should be routinely expanded 2 weeks after catheter removal, and the situation determines the frequency of the dilation. Early postoperative urethral dilatation is an important measure to prevent and treat urethral stricture. Most children with urethral stricture were cured after urethral dilatation. Only some patients with severe stenosis and one patient who also had urethral diverticulum needed another surgery.

Satisfaction of the family members with the appearance of the child’s penis after urethroplasty is also a goal pursued by our urologists. We completely removed ventral fibrosis during the operation, and if necessary, the penile dorsal folding method was used to completely correct chordee. During follow-up, there were no cases of recurrent chordee in our department for nearly 10 years. The PPPS questionnaire is widely used to assess postoperative penile appearance scores [7, 8, 12]. We used the PPPS questionnaire to assess the parents’ satisfaction with the appearance of the penis. The parents’ satisfaction rates with the urinary meatus, glans appearance, shaft skin appearance and general appearance were 85.2, 87.7, 92.3 and 89.0%, respectively. This shows that the parents’ overall satisfaction rate was high, and a satisfactory appearance evaluation can be obtained through the one-stage operation.

This study has some limitations. First, this is a single-centre study with a small sample size. Therefore, further multicentre studies with larger sample sizes are required. Second, the study was a retrospective study without a control group.

## Conclusions

Proximal hypospadias is a serious condition that is often combined with severe chordee, and transverse preputial island flap urethroplasty for single-stage correction is an effective surgical procedure for treating this condition. During the operation, more attention should be given to protecting the blood supply of the vascular pedicle in the foreskin, completely correcting chordee and using rubber drainage strip. The use of postoperative care and disinfection should also be strengthened.

## Data Availability

The datasets used and/or analysed during the current study are available from the corresponding author upon reasonable request.

## Abbreviations

PPPS: Pediatric Penile Perception Score
TPIF: Transverse preputial island flap
